# Functional Trait-Based Evidence of Microplastic Effects on Aquatic Species

**DOI:** 10.3390/biology12060811

**Published:** 2023-06-02

**Authors:** M. Berlino, G. Sarà, M. C. Mangano

**Affiliations:** 1 Stazione Zoologica Anton Dohrn, Department of Integrative Marine Ecology (EMI), Sicily Marine Centre, Lungomare Cristoforo Colombo (Complesso Roosevelt), 90149 Palermo, Italy; mariacristina.mangano@szn.it; 2 Dipartimento di Scienze della Terra e del Mare, DiSTeM, Università degli Studi di Palermo, Ed. 16, 90128 Palermo, Italy

**Keywords:** microplastics, aquatic organisms, functional traits, meta-analysis, effects size

## Abstract

**Simple Summary:**

The potential risks posed by microplastics is fully recognised by the scientific community. Because of the multiple pathways that allow microplastics to reach aquatic ecosystems, researchers have focused their work on the ingestion and impact of microplastics on aquatic organisms. However, the main mechanisms through which microplastics shape ecological responses at different levels in the ecological hierarchy remain understudied, and a high degree of data fragmentation exists in the literature. Functional traits, having indirect effects on the three components of individual fitness: growth, reproduction, and survival, represent the main door through which anthropogenic disturbance can enter, impacting the ecological hierarchy, community structure and composition, and ecosystems’ features. In light of this, this meta-analysis aims at using data available in the literature to understand and assess how the impacts from microplastics spread across the ecological hierarchy, from the individual to the ecosystem level, and how and if microplastics pollution is negatively affecting biodiversity, ecosystem functioning, and the provision of ecosystem goods and services. Developing trait-based indicators represents a useful step to investigate the impacts of microplastics on ecosystems, and at the same time, could be used to guide policy makers in the development of adequate management plans capable of safeguarding ecosystems, together with the valuable goods and services they offer.

**Abstract:**

Microplastics represent an ever-increasing threat to aquatic organisms. We merged data from two global scale meta-analyses investigating the effect of microplastics on benthic organisms’ and fishes’ functional traits. Results were compared, allowing differences related to vertebrate and invertebrate habitat, life stage, trophic level, and experimental design to be explored. Functional traits of aquatic organisms were negatively affected. Metabolism, growth, and reproduction of benthic organisms were impacted, and fish behaviour was significantly affected. Responses differed by trophic level, suggesting negative effects on trophic interactions and energy transfer through the trophic web. The experimental design was found to have the most significant impact on results. As microplastics impact an organism’s performance, this causes indirect repercussions further up the ecological hierarchy on the ecosystem’s stability and functioning, and its associated goods and services are at risk. Standardized methods to generate salient targets and indicators are urgently needed to better inform policy makers and guide mitigation plans.

## 1. Introduction

The accumulation and fragmentation of plastics are recognized worldwide to be threatening our Planet, having detrimental effects on global biodiversity and ecosystem functioning in both terrestrial and aquatic ecosystems [1]. The main mechanisms through which microplastics shape ecological responses at different levels in the ecological hierarchy remain understudied and a high degree of data fragmentation exists in the literature [2,3]. Since mass production of plastic began, plastic waste started to accumulate in terrestrial environments and close to the coasts, reaching oceans and even the most remote environments, including deep-sea beds [4,5,6]. It now represents one of the most pervasive impacts ever recorded. Microplastics—or rather plastic particles of <5 mm in size derived from the fragmentation of macroplastics or manufactured for different applications (i.e., cosmetic, industry) [7]—are spread out in all aquatic systems, including both freshwater and marine habitats [8]; beaches [9]; sediments [10]; and water columns [11]. Greater awareness of the microplastic issue has ensured their inclusion within the Zero pollution action plan of the European Commission [12], which aims to accelerate the transition to a Circular Plastic Economy and a 50% reduction in the plastic litter at sea and 30% reduction in microplastics released into the environment by 2030. Recognizing the potential ecosystem risks posed by microplastics [13], the European Commission (EC) has launched a call for evidence to describe microplastic-related issues with the ambitious objective to tackle microplastic pollution. Furthermore, the EC recognizes the need to quantify the environmental impact at an economic level by promoting the use of ecosystem accounting techniques (SEEA) [14], giving a measure of the potential damage of microplastics on an ecosystem’s ability to provide goods and services.

The potential role of microplastics in impacting aquatic ecosystems, marine species, and food webs has been of particular interest to the scientific community [15,16], which has mainly focused on ingestion [17,18,19,20] or the ecotoxicological effects of microplastic and adsorbed additives [21,22]. However, despite the growing awareness of the problem, microplastic research still lacks standardised protocols for studying their impact on aquatic organisms [23]. This is mainly due to differences in the methods used in conducting the analyses, related to the selection of microplastics with different physical (i.e., shape and size) and chemical (i.e., type of polymer) properties, as well as differences in the experimental design in terms of administration method (i.e., via food, sediment, water), duration of the experiments, and model organism chosen [23,24]. To date, exploring patterns and processes in functional traits is the most recognised way to increase our understanding of how ecological mechanisms respond to both anthropogenic and environment-driven changes in biodiversity and ecosystem functioning [25]. Functional traits represent the main door through which anthropogenic disturbance can enter, impacting the ecological hierarchy (to the upper levels), community structure and composition, and an ecosystem’s features [26]. Functional traits are defined as morphological, physiological, behavioural, and phenological features indirectly impacting fitness through effects on the three components of individual fitness: growth, reproduction, and survival [27,28]. This paper analyses the different responses in aquatic vertebrates’ [2] and invertebrates’ [3] functional traits as a result of the presence of microplastics. We analysed the effect of microplastics relative to ecological factors (i.e., habitat, life stage from larvae to adult, trophic level) and to the experimental design adopted in each study (i.e., microplastic size, type, shape, and days of exposure). The information yielded helps us better understand and assess how the impacts from microplastics spread across the ecological hierarchy, from the individual to the ecosystem level, and how and if microplastic pollution is negatively affecting biodiversity, ecosystem functioning, and the provision of ecosystem goods and services.

## 2. Materials and Methods

### 2.1. The Literature Search and Data Collection

The data used for this meta-analysis derive from two previously published papers [2,3]. The two databases were merged with the aim of obtaining a single matrix that would allow for a comparison of the results, providing a measure of the potentially different degrees of impact of microplastics on the two aquatic compartments. This comparison is maintained throughout the meta-analytical process further highlighting variation in the measured outcomes and disentangling the guiding factors of the measured effect sizes. Collection of the papers was performed through a literature search [29,30], with the aim to answer our main question “What are the impacts of microplastics on the functional traits of aquatic organisms?”. The two main academic literature databases, ISI Web of Knowledge (Web of Science Core Collection package, Clarivate Analytics, 2019) and Scopus were used to search for the peer-reviewed literature, without any limitation on the temporal scale. In doing so, we created a complex search string including the main elements of our primary question linked by the Boolean operator “AND” and all synonyms linked by the Boolean operator “OR” [29,30]. These four elements are represented by: the exposure (i.e., microplastic with related keywords), the measured outcomes (i.e., traits and related keywords), the target population (the subject of the search, i.e., benthic organisms or fish) and the observation type (e.g., laboratory, mesocosm, and related terms). Specifically, the last groups of keywords were added to prevent the inclusion of a high number of studies with no report of associated measured effects of microplastic on aquatic organisms or focusing their attention on the occurrence of microplastics in the environment. The measured outcomes, the list of traits considered, and associated description/quantification, and the variables measured (keywords) in the selected studies are provided in Table S1.

We considered species from all the aquatic spheres, therefore inhabiting freshwater, marine, and estuarine environments, and from different life stages (i.e., larvae, juvenile, and adult) with the aim to test the global effect of microplastics on aquatic organisms. We selected studies testing the response of experimental treatment groups (i.e., organisms exposed to microplastics) against one or more untreated control groups (i.e., organisms not exposed to microplastics). Moreover, all the studies included had to report the mean values for the functional trait investigated and the variability around the mean of the measured variables together with the sample size used during the experiments (Table S2). We included studies that focused on the effects of microplastics on the functional traits of aquatic organisms that are measurable at individual levels, as well as variables that had direct effects at population levels. The functional traits we focused on were divisible into three main groups: morphological (e.g., body length), physiological (e.g., hepatosomatic index, respiration) and behavioural (e.g., swimming activity) [27,28]. These traits are usually involved in optimising individual fitness and have implications scaling up to the population level, such as changes in growth and mortality response. For those studies in which mortality rate was reported, these values were converted into survival rate where possible, so that the measures reported for this category were survival values only. Therefore, all observational studies were excluded, i.e., studies assessing the presence and concentration of ingested microplastic polymers, studies that used pollutants or other chemical compounds (e.g., antibiotics) added to the microplastics, as well as studies that focused on the effects at sub-organismal level (e.g., examining cellular and subcellular variables such as oxidative stress, gene expression, immunological responses, etc.). Reviews, experimental treatments focused on the effects of nanoplastics papers that were not based on the study of functional traits of aquatic organisms, or studies not containing sufficient data were also excluded. The selection criteria ensured that only papers with a clear description of the experimental design, such as comparisons of experimental treatment groups with one or more control group (i.e., a group of organisms exposed—“treated”—to microplastics tested against unexposed organisms—“untreated”), were included in the meta-analysis. The scientific papers that were deemed appropriate for our analysis were merged resulting in a final global dataset of 82 scientific papers.

### 2.2. Calculation and Analysis of Effects

To account for the different variables and approaches in the selected scientific papers, we used Hedges’ *g* statistic to estimate the difference in microplastic effects between an experimental treated group and a control group. Hedges’ *g* is the bias-corrected standardized mean difference between the treatment and control groups, divided by the pooled standard deviation [31,32]. Hedges’ *g* value and its variance were calculated for each case study (*k* = 1473 case studies in our dataset). Hedges’ *g* weighs cases by their sample size and the inverse of their variance [33] The value of Hedges’ *g* ranges from −∞ to +∞ and can be interpreted as follows [34]: |*g*| ≤ 0.2 is considered a small effect; 0.2 ≤ |*g*| ≤ 0.5 is considered a medium effect; 0.5 ≤ |*g*| ≤ 0.8 is considered a large effect; and |*g*| ≥ 0.8 is considered a very large effect. Hedges’ *g* effect size was calculated as follows [33]:

$${\mathrm{Hedges}}^{’}g=\frac{\left(\bar{Y}t-\bar{Y}c\right)}{\mathrm{standard}\;\mathrm{deviation}\;\mathrm{pooled}}\times \;J$$

where *Yc* and *Yt* are the mean values of the control and experimental treatment groups, respectively.

Correction for bias due to different sample sizes, represented by *J*, was estimated by differentially weighting the studies as follows:

$$J=1-\frac{3}{4\left(N_{t}+N_{c}-2\right)-1}$$

While the following formula was used to calculate the pooled standard deviation (standard deviation pooled):

$$standard\;deviation\;poole=\sqrt{\frac{\left(N_{t}-1\right)\times s.d._{t}{}^{2}+\left(N_{c}-1\right)\times s.d._{c}{}^{2}}{N_{t\;}+N_{c}-2}}$$

where *N* is the sample size, and *s.d.* is the standard deviation of the treated group and control group, respectively. To account for inequality in study variance, effect sizes were weighted by the inverse of the sampling variance, therefore calculating variance for each effect size (*V_g_*) as follows [34]:

$$V_{g}=\frac{N_{t}+N_{c}}{n_{t}n_{c}}+\frac{g^{2}}{2\left(n_{t}n_{c}\right)}$$

Since the sign of Hedges’ *g* indicates the direction of the effect, a negative value of Hedges’ *g* shows that microplastics have a higher effect on that specific response analysed.

To measure the magnitude of the effect size on individual response variables and to minimize the large heterogeneity of the dataset, we used behaviour, metabolism, growth, reproduction, and survival as the five response categories (Table S1). Therefore, we ran a model to estimate the overall effect size and 95% CI per category.

Differences in the pooled effect size among the tested variables correlated with the biology and ecology of aquatic organisms or the experimental conditions of exposure to microplastics, and were tested by performing subgroup analysis including the following fixed factors as moderators of the mixed-effects model: habitat (freshwater, marine, and estuarine); life stage of aquatic organism (larvae, juveniles, adults); trophic level (level 1) consisting of all organisms that feed on plant or algae and those who actively obtain organic matter from the abiotic matrix (water and sediment); level 2 consumers are represented by organisms that ingest prey); microplastic type (different effects depending on the use of a mixture of microplastics or different types of polymers); microplastic shape (divided into fibres, fragments, spheres); microplastic size (<25 µm, 25–100 µm, 100–500 µm, >2000 µm); and duration of exposure (from less than 1 day to more than 90 days) (Table S2). Ultimately, we performed meta-regression analysis with mixed-effects model using microplastic concentration as continuous fixed factor to investigate the possible correlation between the concentration of microplastics used in the experiment and their effects on functional traits of aquatic organisms. In order to obtain suitable data for the application of the model and face the heterogeneity in the methodology applied by different authors (i.e., sample design and different units to express MPs concentration), we divided our dataset into three main groups concerning the medium used for microplastic administration (i.e., water, sediment or food). Then, we used these three main groups to identify the principal method for measuring concentration (i.e., number of particles, weight of the particles or percentage of the particles in reference to the medium used for administration) and therefore standardized the unit concentration in order to have comparable data for each group. Given the high concentration used in some of the case studies included in our meta-analysis, we tested multiple models on the same group first with the total number of case studies and then removed those studies with higher concentrations than environmentally recorded. Table S3 in Supplementary Materials reports the number of case studies for each group and summarizes the above in a schematic way, while Table S4 reports results for all the models.

The meta-analyses were conducted using the metafor package for R [35,36]. We performed mixed effect models using the ‘rma.mv’ function, which uses a Wald-type test to determine statistical significance. We ran a statistical model that included the study’s identification number (i.e., ID of the study in our dataset) and the response variable (i.e., functional trait categories) as a random factor to account for heterogeneity [37] and non-independence of results from the same study [2,3,38]. 

Effect size for the models including categorical fixed factor was considered to be significant if their 95% CI (Confidence Interval) did not overlap with zero and if their *p*-values were ≤0.05. For the model with a continuous fixed factor (i.e., concentration of microplastic), the predictor was considered to be significant at *p* ≤ 0.05. Differences between the groups included as moderators in the subgroup analysis were considered to be significant when the *p*-value of the test for moderators (*Qm*) calculated in the mixed-effects model was ≤0.05. Results from different models were compared by running a fixed-effects meta-regression using estimates and standard errors (i.e., effect size of the model) from each model and including categorical variables identifying the different model estimates as moderators [39].

## 3. Results

### 3.1. Overall Analysis 

**Figure 1:**
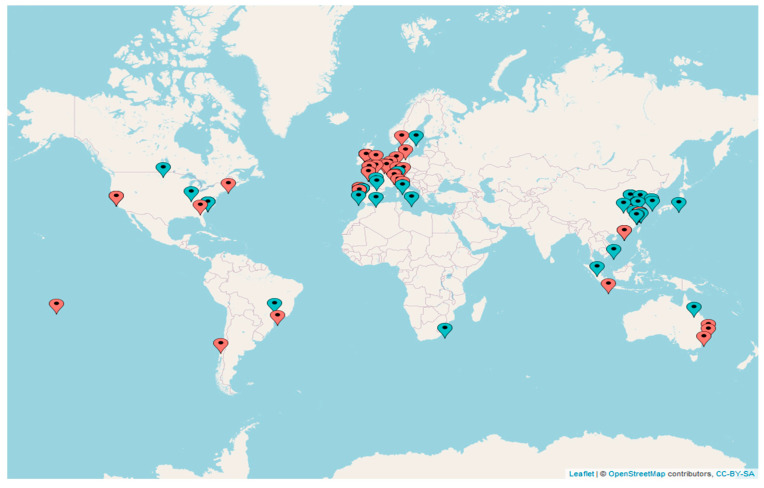
Geographical map reporting global distribution of case studies. Blue pins indicate studies on fish; orange pins indicate studies on benthic organisms. An interactive online version reporting authors’ names and dates of publication, species’ scientific name, life stage, and functional traits studied in each scientific paper is available at https://mberlino.github.io/mberlino-github.io/ (accessed on 20 July 2022).

The previous extensive literature reviews [2,3] provided us with a global dataset (see map of the studies’ global distribution in Figure 1), which consisted of 82 selected papers accounting for a total of 1473 (*k* = 1473) case studies. The dataset included almost one thousand case studies (*k* = 831) for benthic organisms and 642 case studies for fish (details of the included studies, i.e., the number of case studies and associated variables, are reported in Table S2, Supplementary Materials).

**Figure 2:**
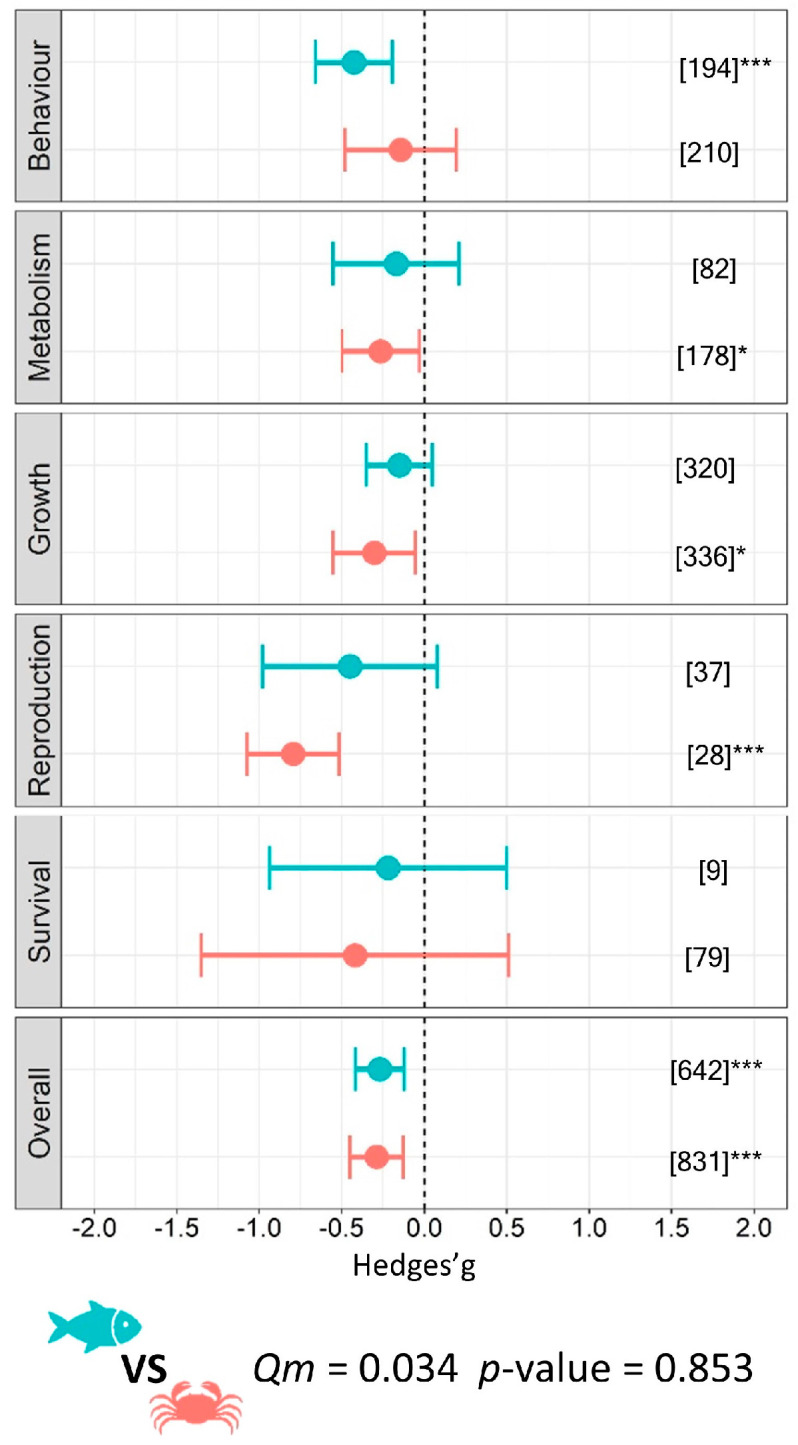
Effect of MPs on the functional traits of aquatic organisms. Circles and horizontal lines represent Hedges’ *g* value and 95% CI for each effect size, respectively; blue circles indicate results for fish and orange circles for benthos; number of case studies (*k*) specified in brackets. Icons report comparison between fish and benthos model. Levels of statistical significance: *p* ≤ 0.001 ***, *p* ≤ 0.01 **, *p* ≤ 0.05 *. Mixed-effects model was performed using the rma.mv function of the metaphor package in R, including study ID and functional trait as a random factor.

Results obtained using a mixed-effects model on the entire database (*k* = 1473) confirmed an overall negative impact on functional traits from microplastics for all analysed aquatic organisms (*g* = −0.28 ± 0.11; *p* < 0.001) showing a medium effect size (benthos *g* = −0.29 ± 0.16, *p* = 0.001; fish *g* = −0.27 ± 0.15, *p* < 0.001, respectively) although no statistical difference occurred when comparing the two overall effects (*Qm =* 0.034, *p* = 0.853). Nevertheless, a significant effect size for benthic organisms and fish was found for different individual response variables. Accordingly, metabolism (*g* = −0.26 ± 0.24, *p* = 0.028), growth (*g* = −0.30 ± 0.25, *p* = 0.017), and reproduction (*g* = −0.80 ± 0.28, *p* < 0.001), were significantly affected by microplastics in the benthic organism, while only the variable ‘behaviour’ was significant in the case of fish (*g* = −0.43 ± 0.23, *p* < 0.001). We did not detect differences for the other functional traits investigated in this study (Figure 2).

### 3.2. Subgroup Analysis

**Figure 3:**
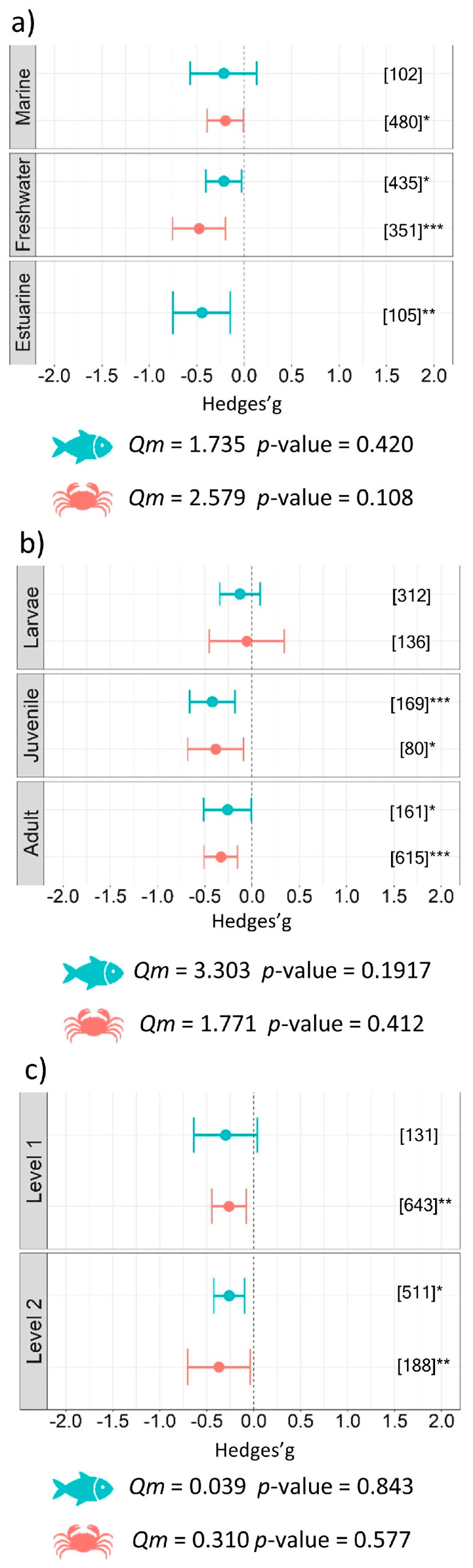
Results of subgroup analysis with mixed effect model for (**a**) habitat, (**b**) life stage, and (**c**) trophic levels of aquatic organisms. Circles and horizontal lines represent Hedges’ *g* value and 95% CI for each effect size, respectively; number of case studies (*k*) specified in brackets. Icons reports *Qm* = omnibus test of moderators from the model. Levels of statistical significance: *p* ≤ 0.001 ***, *p* ≤ 0.01 **, *p* ≤ 0.05 *. Mixed-effects model was performed using the rma.mv function of the metaphor package in R, including study ID and functional trait as a random factor.

Investigating differences according to the habitat of aquatic organisms revealed that freshwater species were more impacted (benthos *g* = −0.48 ± 0.28, *p* < 0.001; fish *g* = −0.21 ± 0.19, *p* = 0.025) than estuarine fishes (*g* = −0.45 ± 0.30, *p* = 0.004) and marine benthic species (*g* = −0.20 ± 0.19, *p* = 0.043). The effect on marine fish was not significant (*g* = −0.22 ± 0.35, ns) (Figure 3a). Our subgroup analysis, including life stage as a moderator, supported a more negative effect on juveniles of aquatic benthic organisms (*g* = −0.38 ± 0.30, *p* = 0.011) and fish (*g* = −0.42 ± 0.24, *p* < 0.001) than adults. No significant effect was detected within larval life stages in either the benthic or fish datasets (Figure 3b). Trophic level 2 showed a significant result for both fish (*g* = −0.26 ± 0.16, *p* = 0.002) and benthos (*g* = −0.37 ± 0.33, *p* = 0.028), while only benthic organisms in trophic level 1 (*g* = −0.26 ± 0.18, *p* = 0.005) were impacted by microplastics (Figure 3c). Subgroup analysis looking at possible differences related to the biology and ecology of aquatic organisms did not identify significant differences between groups included as moderators in the model (i.e., habitat, life stage and trophic level, Table 1).

**Figure 4:**
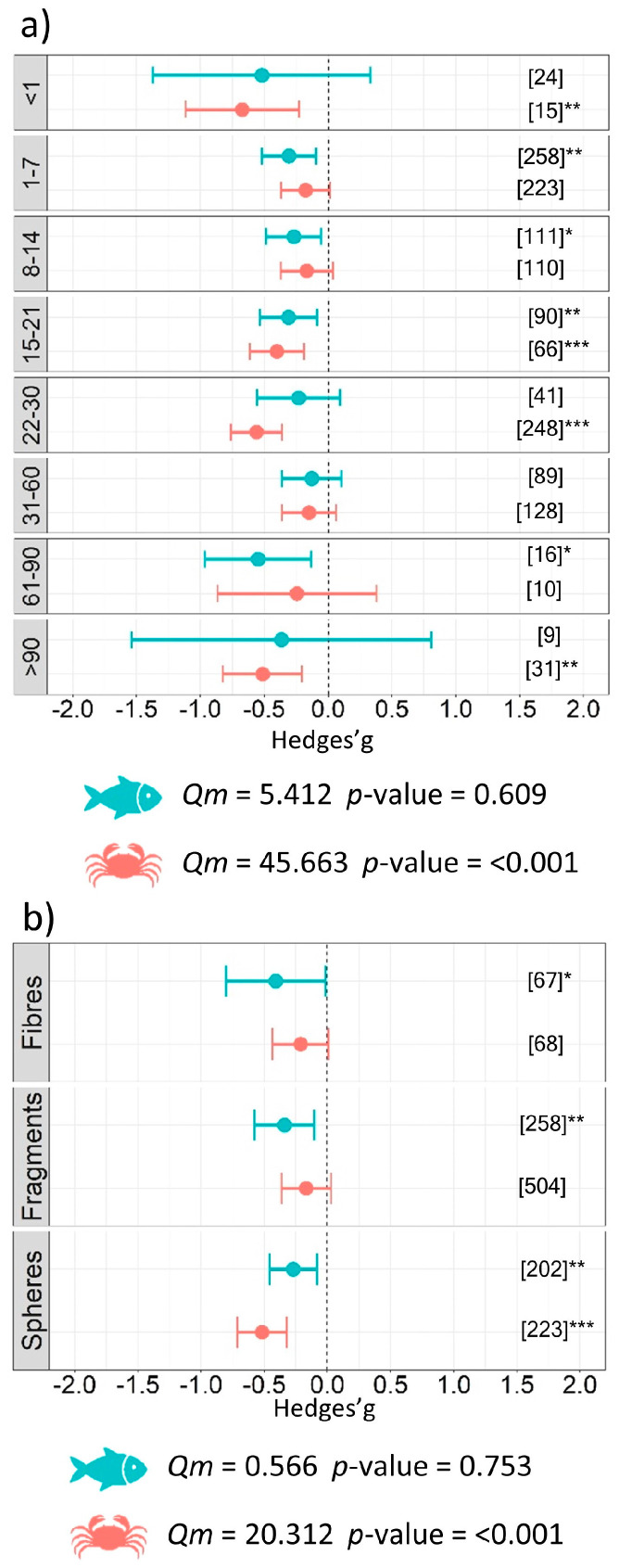
Results of subgroup analysis with mixed effect model for (**a**) duration of the experiment (days), and (**b**) shape of microplastics used in the experiments. Circles and horizontal lines represent Hedges’ g value and 95% CI for effect size, respectively; number of case studies (*k*) specified in brackets. Icons report *Qm* = omnibus test of moderators from the model. Levels of statistical significance: *p* ≤ 0.001 ***, *p* ≤ 0.01 **, *p* ≤ 0.05 *. Mixed-effects model was performed using the rma.mv function of the metaphor package in R, including study ID and functional trait as a random factor.

**Figure 5:**
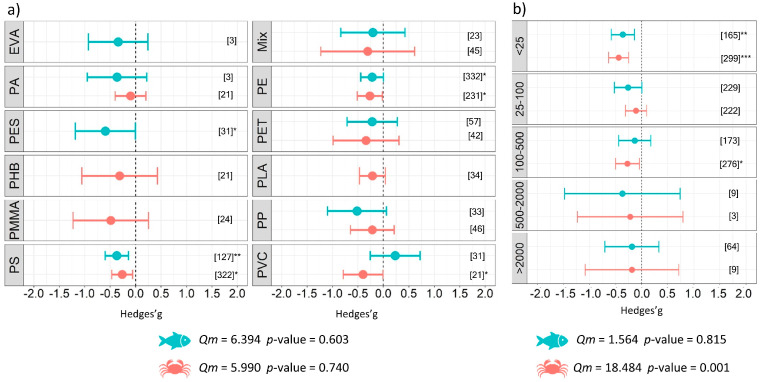
Results of subgroup analysis with mixed effect model for (**a**) type, and (**b**) size (µm) of microplastics used in the experiments. Circles and horizontal lines represent Hedges’ g value and 95% CI for effect size, respectively; number of case studies (k) specified in brackets. Icons report Qm = omnibus test of moderators from the model. Levels of statistical significance: *p* ≤ 0.001 ***, *p* ≤ 0.01 **, *p* ≤ 0.05 *. Mixed-effects model, using the rma.mv function of the metaphor package in R, including study ID and functional trait as a random factor.

The analysis conducted on the components of the experimental design, specifically experiment duration, revealed that exposure to microplastics in a temporal range between 1 and 90 days resulted in a negative impact with differences between the fish and benthos. A significant negative effect was observed for short durations (less than one day) of exposure to microplastics (*g* = −0.67 ± 0.45, *p* = 0.003) for benthic organisms. When the experiment was longer than 22 days and up to 30 days, the effect size in experiments involving benthos was significant (*g* = −0.56 ± 0.20, *p* < 0.001). The same pattern was found for fish when they were exposed to microplastics in experiments ranging between 1 and 7 days of exposure (*g* = −0.31 ± 0.21, *p* = 0.005) and between 61 and 90 days (*g* = −0.55 ± 0.42, *p* = 0.010) (Figure 4a). The smallest microplastic size class (i.e., <25 µm) significantly affected both categories of organisms as follows: *g* = −0.44 ± 0.19, *p* < 0.001 for benthos, and *g* = −0.36 ± 0.22, *p* = 0.001 for fish. Significant results were also obtained for size class: 25–100 µm (*g* = −0.26 ± 0.26, *p* = 0.054) for fish, and size class 100–500 µm (*g* = −0.27 ± 0.23, *p* = 0.019) for benthos (Figure 5b). Subgroup analysis investigating the shape of polymers showed that spheres (*g* = −0.52 ± 0.20, *p* < 0.001) and fragments (*g* = −0.34 ± 0.24, *p* = 0.005) represented a major issue for benthos and fish, respectively (Figure 4b). No significant differences were detected between the groups of microplastic type for fish (*Qm* = 6.40, *p* = 0.603) or benthos (*Qm* = 6.00, *p* = 0.740); however, our results demonstrate a negative impact from the commonly used polyethylene (PE) and polystyrene (PS) microplastics on aquatic organisms (Figure 5a details the estimates for each type of microplastic used). Finally, the subgroup analysis conducted on the experimental conditions revealed significant differences between groups of microplastic shape (*Qm* = 20.31, *p* <0.001), days of exposure to microplastics (*Qm* = 45.66, *p* < 0.001), and size (*Qm* = 18.48, *p* = 0.001) for benthic organisms, while no differences among groups were found for fish (Table 2).

**Figure 6:**
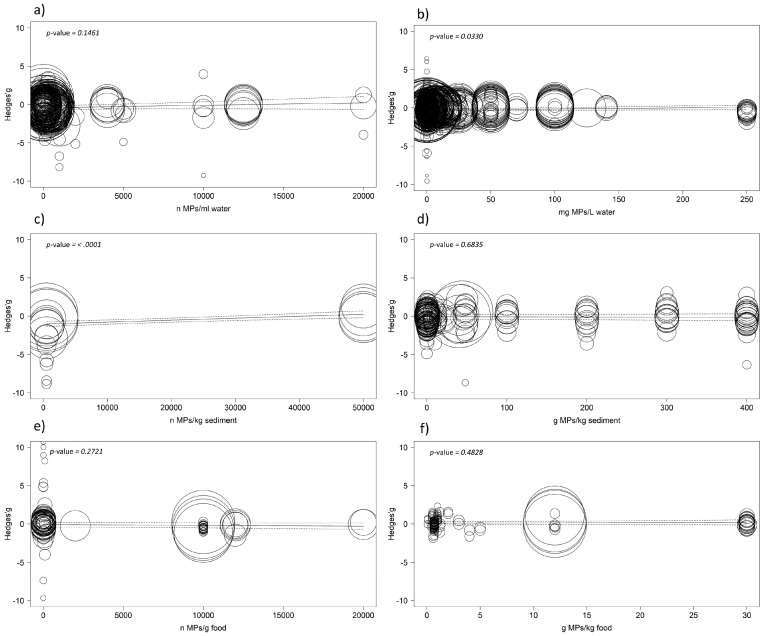
Meta-regressions showing relation of effect size and microplastic concentration in water (**a**,**b**), sediment (**c**,**d**), and (**e**,**f**) food. Mixed-effects model was performed using the rma.mv function of the metaphor package in R, including study ID and functional trait as random factor. The size of the point is related to the standard error of the study; dotted lines indicate 95%.

Results of the meta-regressions did not reveal any negative effects on the functional traits of aquatic organisms related to the concentration of microplastics, except when looking at the number of microplastics per litre of water at the highest concentration (*p* = 0.010). When removing this case study, no significant effects were detected (*p* = 0.146). Looking at the concentration expressed in milligrams of microplastics per litre, results show a significant but positive correlation (*p* = 0.033), meaning the effect size increases with concentration and therefore leads to a not-negative effect on functional traits. The model also returned highly significant results with a positive correlation (*p* < 0.001) for the number of microplastics per kilogram of sediment but there was no significant result for grams of microplastic in kilograms of the same medium (*p* = 0.683). Concerning microplastics administrated with food, results are not significant for both concentrations expressed as the number of microplastics per kg (*p* = 0.272) and grams of microplastics per gram (*p* = 0.482) of food (Figure 6).

## 4. Discussion

Our global meta-analysis confirmed the negative impact of microplastics on aquatic vertebrates’ and invertebrates’ functional traits. The synthesis of the collated scientific evidence showed that microplastics are ingested independently by vertebrates and invertebrates [40,41,42,43] and, more interestingly, that regardless of the habitat (freshwater, marine, and estuarine), their functional traits are negatively affected. These findings sound an alarm for the Earth’s whole aquatic sphere [44,45], revealing an unprecedented level of impact that spans from invertebrates to vertebrates, potentially affecting over 90% of the world’s aquatic biodiversity. When the effects of microplastics are examined through functional traits, the results show that the entire ecological hierarchy may be impaired, and thus, overall ecological performance can be affected, setting in motion a chain of events that can undermine ecosystem functioning [46,47] and the provision of goods and services [48]. Both fishes and benthos showed an overall medium effect size, yet their specific responses were different when looking at single response variables (Figure 2). For example, traits related to energy consumption, allocation, and assimilation (i.e., metabolism) were significantly affected in invertebrates and not affected in fish. The cause of this difference is not straightforward, although we can hypothesize that feeding behavioural traits are a key driver. Benthic organisms included in our dataset are mainly scavengers and deposit-feeders, relying on sediments, thereby they manipulate sedimentary materials to get food or exploit sediments for refuge [49,50]. Such behaviours, when translated into the natural environment, would increase the likelihood of encountering and ingesting microplastics trapped in sediments, which are the main sink of microplastics in aquatic habitats worldwide [44]. The potential food dilution resulting from the presence of microplastics [51] can (i) directly affect the amount of acquired and assimilable energy during feeding [52,53], and (ii) indirectly cause secondary effects on other behavioural traits such as searching for food [54], which can have repercussions on the half-saturation constant of the functional response [55]. Overall, this leads to a general impact on an organism’s energy budget [56], generating conditions of energy depletion, through alterations to the functioning of the metabolic machinery with consequent effects on individual fitness and life history traits (e.g., survival, growth, reproduction etc.).

Similarly, microplastics may affect behavioural traits in fishes but through different pathways. Even when not ingested, microplastics can affect fishes’ behaviour. For example, when they adhere to gills and skin, this impairs oxygen flow and ion regulation, which in turn causes respiratory stress; therefore, there are potential resultant impacts on general behaviours such as swimming, foraging, and mating [57,58]. However, when mixed with food, plastic particles can be mistaken as prey and ingested [59], causing negative effects on feeding capacity and gastrointestinal blockages [60,61]. The accumulation of microplastics in the digestive tract can lead to malnutrition and eventually starvation [62], which means that less energy and resources are available for the fundamental physiological processes of growth and reproduction [51]. Fish that receive only a small amount of energy may become less responsive and slower at swimming. If they suffer intestinal injuries, the fish may exhibit abnormal swimming patterns [63]. In addition, impaired locomotion can have a significant impact on fish as prey or predator, affecting their survival (higher predation) or growth rate (feeding efficiency), and leading to population declines [64].

In addition to the effects demonstrated on the individual, it is necessary to focus on those at the ecosystem level, given that all the aforementioned impacts on functional traits can occur simultaneously in both fish and benthic species. Microplastics may affect population dynamics and trophic interactions, easily impairing ecosystem stability and functioning [47]. The effects on the population could directly or indirectly affect ecological and specifically trophic interactions, i.e., the core functions of any ecological system [65], for example, impacting predator–prey relationships. We detected trophic effects in our investigation. For example, while level 2 consumers were impacted in both the fish and benthos dataset, only in benthic organisms level 1 consumers were significantly affected. This could be explained by the behavioural traits involved in feeding and the close association of benthic organisms with sediments, which increases the likelihood of benthos encountering microplastics compared to fishes that more frequently exploit the upper layers of the water column. Furthermore, this has important ecological repercussions, as level 1 consumers are often the prey for level 2 consumers, especially for predators and benthivores. Given that the functional traits of level 1 consumers are impaired by microplastics with consequences for individual fitness and population dynamics, there will be a subsequent reduction of available prey for level 2 consumers. Predator–prey interactions are therefore impacted, impairing the energy transfer through entire trophic webs. Furthermore, microplastics may move through food webs and impair higher trophic levels [66,67].

Analysis conducted on experimental conditions revealed how the microplastics’ size and shape represent the most important factors affecting functional traits. Indeed, results for meta-regressions on microplastic concentration showed a significant negative effect only at an extremely high concentration, and while removing these case studies the results turn out to be positively correlated or not significant. Microspheres appeared to have a larger impact on both fish and benthos functional traits, while a regular shape of spheres seemed to enhance the possible transport and translocation of the particles through the digestive apparatus [58]. As regular-shaped polymers are usually adopted in manipulative experiments [68], the results cannot be generalised to a natural setting; this is because fibres and fragments in natural environments are irregularly shaped and, as reported in other studies, they seem to have a greater impact because of their rough surface (sharp edges) causing more physical damage [69].

Regarding the dimension of microplastics, smaller particles were found to have a more negative effect. Microplastics of smaller size could be ingested by a greater number of organisms belonging to many different species, amplifying the likelihood that the particles would be channelled through the food web [70]. Organism size, and thus the life stage, is a key factor in interpreting these results. Larvae that develop slowly, and whose mouthparts are not fully developed or too small, may not be able to swallow microplastic items that are larger than their mouth, and, therefore, are not exposed to specific size ranges of microplastics [71,72,73]. Subgroup analysis revealed that the juvenile life stage was the most impacted group in both fish and benthos. This is in line with results from our previous work [2], where we found a significant correlation between increasing larval size and negative impact. This suggests that the negative effect of microplastics increases when individuals reach the juvenile life stage and have developed their feeding abilities, allowing them to ingest a wider range of particles.

## 5. Conclusions

Microplastics can be ingested by all aquatic organisms leading to an overall negative impact, however, the results of this work show that there are differences in terms of effects on the functional traits of fish and benthic organisms. While MPs mainly affect the behaviour of the former, invertebrates suffer from a negative effect to the detriment of metabolic functions, impacting their energy budget and consequently individual fitness. The analysis further demonstrates how organismal responses differed by trophic level with the level 1 consumers being negatively impacted only when analysing benthic organisms. Feeding behaviours of organisms associated with sediments could lead to more encounters of MPs in the natural environment and lead to important ecological consequences due to the trophic transfer of microplastics and to alteration of prey–predator interactions. Our work highlights the strong heterogeneity of experimental design, which aside from making data collection and extraction more difficult, precludes the possibility of analyzing some other important factors such as the concentration and density of microplastics. This is a serious drawback; especially as political institutions are currently showing an interest in scientific evidence that measures the impacts of microplastics on the ecosystem. There is a clear need for a mandatory standardization of methods, that would support experiment replicability around the globe and further investigations into the aspects that may influence the performance of organisms across the food web (biomagnification and bioaccumulation) [74]. Our results, keeping in mind the definition of functional traits proposed by Violle et al. [28], highlight the importance of focusing on functional traits to identify potential impacts on organisms, suggesting that one of the most important aspects to be treated as a first stepping stone would be to focus future studies on the functional traits that mainly affect individual performance and ultimately individual fitness (i.e., growth, reproduction, and survival) with a view to being able to use the effects measured at the lowest level of the ecological hierarchy to predict the possible repercussions that could ultimately affect the stability of aquatic ecosystems. Developing trait-based indicators [75] represents a necessary step to obtain sound results. These indicators would be useful for investigating impacts on ecosystems, and at the same time, could be used to guide policy makers in the development of adequate management plans capable of safeguarding ecosystems, together with the valuable goods and services they offer.

## Figures and Tables

**Table 1:** Test of moderator for variables related to the biology and ecology of aquatic organisms. Summary of the results of the omnibus test of moderators (*Qm*) for each model; df = degree of freedom; *p*-values: statistical significance of the test.

Variable	Dataset	*Qm*	df	*p*-Value
Trait	Fish vs. Benthos	0.034	/	0.853
Habitat	Fish	1.735	2	0.420
	Benthos	2.579	1	0.108
Life stage	Fish	3.303	2	0.191
	Benthos	1.771	2	0.412
Trophic level	Fish	0.039	1	0.843
	Benthos	0.310	1	0.577

**Table 2:** Test of moderator for variables related to the experimental condition of the studies. Summary of the results of the omnibus test of moderators (*Qm*) for each model; df = degree of freedom; *p*-values: statistical significance of the test.

Variable	Dataset	*Qm*	df	*p*-Value
Type	Fish	6.394	8	0.603
	Benthos	5.990	9	0.740
Size	Fish	1.564	4	0.815
	Benthos	18.484	4	0.001
Duration	Fish	5.412	7	0.609
	Benthos	45.663	7	<0.001
Shape	Fish	0.566	2	0.753
	Benthos	20.312	2	<0.001

## Data Availability

The data presented in this study are available on request from the corresponding author.

## Supplementary Materials

The following supporting information can be downloaded at: https://www.mdpi.com/article/10.3390/biology12060811/s1, Table S1: Description of the five functional traits examined in the meta-analysis and keywords for the measured variables used in the selected studies for each functional trait category; Table S2: Summary of all the studies included in the global dataset ordered by species name [49,50,58,59,63,73,76,77,78,79,80,81,82,83,84,85,86,87,88,89,90,91,92,93,94,95,96,97,98,99,100,101,102,103,104,105,106,107,108,109,110,111,112,113,114,115,116,117,118,119,120,121,122,123,124,125,126,127,128,129,130,131,132,133,134,135,136,137,138,139,140,141,142,143,144,145,146,147,148,149,150,151]; Table S3: Summary of grouping and case studies number for concentration analysis; Table S4: Summary of model with continuous factor.
