# Supplementary material for: Functional Trait-Based Evidence of Microplastic Effects on Aquatic Species
Source: Biology (Basel). 2023 Jun 2;12(6):811. doi: 10.3390/biology12060811 (PMC10294819; doi:10.3390/biology12060811)
Supplement: Supplementary file 1 [file biology-12-00811-s001.zip › biology-2318098-supplementary.pdf]

SUPPLEMENTARY INFORMATION

FUNCTIONAL TRAIT BASED EVIDENCE OF MICROPLASTIC EFFECTS ON AQUATIC SPECIES

Table S1 **Description of the five functional traits examined in the meta-analysis and keywords for the measured variables used in the selected studies for each functional trait category.** Specifically, the two functional traits – behaviour and metabolism – and the three performance traits – growth, reproduction, survival – as from Arnold's (1983) framework revisited by Violle et al. (2007). Regarding the quantification of variables such as those associated with behaviour, we followed the quantification reported by the authors, according to the methods they applied.

**Functional Trait**	**Description**	**Keywords**
Behaviour	Organisms behavioural and feeding changes reported after the ingestion or exposure to microplastics compared to an unexposed control group	Activity rate, locomotor activity, mobility, swimming velocity, maximum velocity, distance travelled, spontaneous movement, turning behaviour, inactivity, use of the tank, predatory activity, feeding success, foraging time
Growth	Variation in organism size per unit of time, capability to grow specific parts of the organism or complete transformation stages measured after exposure to microplastics compared to an unexposed control group	Body weight, body length, standard length, total length, weight gain rate, body mass, changes in body mass, length-weight ratio, biometric measurements, head length, head height, head depth, liver weight, gill weight, gonad weight, swim bladder area, optic vesicle area, pericardium area, angle between myosepts, distance between myosepts, interocular distance
Metabolism	Metabolic energy expenditure or available as reserves for the individual after the ingestion of microplastics compared to an unexposed control group	Energy consumption, oxygen consumption, assimilation efficiency, macronutrient, condition factor, hepatosomatic index, gonadosomatic index, heart beats, mucus secretion, byssus secretion
Reproduction	Variation in gametes or embryos production or<br>impairment in asexual reproduction after exposure to microplastics compared to an unexposed control group	Oocite production, sperm motility, brood size, embryos production, egg production, fertilization rate
Survival	Survival rate of organisms exposed to microplastics compared to an unexposed control group	Survival rate, survival percentage

Table S2 **Summary of all the studies included in the global dataset ordered by species name.** Country where the experiment took place, species name, habitat, trophic level and life stage of the organisms used are listed together with functional traits and number of case studies reported as *k*.

Authors	Country	Species	Habitat	Trophic level	Life stage	Trait	k
Critchel et al., 2018[1]	Australia	*Acanthochromis polyacanthus*	Marine	Level 1	Juvenile	Behaviour	10
						Metabolism	12
						Growth	33
Rochman et al., 2016[2]	USA	*Acipenser transmontanus*	Estuarine	Level 2	Juvenile	Behaviour	4
						Metabolism	4
Naidoo et al., 2019[3]	South Africa	*Ambassis dussumieri*	Estuarine	Level 2	Juvenile	Growth	9
Jabeen et al., 2018[4]	China	*Carassius auratus*	Freshwater	Level 1	Adult	Growth	6
						Metabolism	3
Choi et al., 2018[5]	Republic of Korea	*Cyprinodon variegatus*	Estuarine	Level 1	Larvae	Behaviour	16
Xia et al., 2020[6]	China	*Cyprinus carpio*	Freshwater	Level 1	Larvae	Growth	3
Chen et al., 2017[7]	China	*Danio rerio*	Freshwater	Level 2	Larvae	Behaviour	21
Duan et al., 2020[8]	China						
Karami et al., 2107[9]	Malaysia					Metabolism	15
Qiang & Cheng, 2019[10]	China						
Malafaia et al., 2020[11]	Brazil					Growth	132
Le Moine et al., 2018[12]	Canada						
Qiao et al., 2019[13]	China				Juvenile	Metabolism	4
						Growth	6
Kim et al., 2019[14]	Republic of Korea				Adult	Behaviour	29
Jin et al., 2018[15]	China					Metabolism	4
Limonta et al., 2019[16]	Italy					Growth	4
Zhao et al., 2020[17]	China						

Mazurais et al., 2015[18]	France	*Dicentrarchus labrax*	Marine	Level 2	Larvae	Behaviour	2
Barboza et al., 2018[19]	Portugal	*Dicentrarchus labrax*	Marine		Juvenile	Metabolism	6
Pedà et al., 2016[20]	Italy	*Dicentrarchus labrax*	Marine		Adult	Growth	16
						Survival	1
Jinhui et al., 2019[21]	China	*Hippocampus kuda*	Marine	Level 2	Juvenile	Growth	14
						Metabolism	7
						Survival	1
Guven et al., 2018[22]	Vietnam	*Lates calcarifer*	Estuarine	Level 2	Larvae	Behaviour	1
					Juvenile	Behaviour	1
Zhu et al., 2020[23]	USA	*Orizias latipes*	Freshwater	Level 2	Larvae	Behaviour	6
Chisada et al., 2019[24]	Japan					Growth	14
Hu et al., 2020[25]	USA				Adult	Growth	40
Pannetier et al., 2019[26]	France					Metabolism	12
						Reproduction	30
Cong et al., 2019[27]	China	*Oryzias melastigma*	Freshwater	Level 2	Larvae	Behaviour	39
Le Bihanic et al., 2020[28]	France					Growth	20
Li et al., 2020[29]	China					Survival	4
Wang et al., 2019[30]	China				Juvenile	Metabolism	12
					Adult	Reproduction	7
Lönnstedt et al., 2016[31]	Sweden	*Perca fluviatilis*	Freshwater	Level 1	Larvae	Behaviour	22
						Growth	2
Malinich et al., 2018[32]	USA	*Pimephales promelas*	Freshwater	Level 1	Larvae	Behaviour	12
					Juvenile	Growth	7
de Sá et al., 2015[33]	Portugal	*Pomatoschistus microps*	Estuarine	Level 2	Juvenile	Behaviour	20
Ferreira et al., 2016[34]							
Fonte et al., 2016[35]						Growth	4
Luís et al., 2015[36]							
Miranda et al., 2019[37]							
Schmieg et al., 2020[38]	Germany	*Salmo trutta*	Freshwater	Level 2	Juvenile	Growth	1

Yin et al., 2019[39]	China	*Sebastes schlegelii*	Marine	Level 2	Juvenile	Behaviour	11
Yin et al., 2018[40]						Metabolism	3
Espinosa et al., 2017[41]	Spain	*Sparus aurata*	Marine	Level 2	Adult	Growth	8
Wen et al., 2018[42]	China	*Symphysodon aequifasciatus*	Freshwater	Level 2	Juvenile	Growth	2
						Survival	2
Green et al., 2016[43]	Northern Ireland	*Arenicola Marina*	Marine	Level 1	Adult	Behaviour	9
van Cauwenberghe et al., 2015[44]	Belgium					Metabolism	14
						Growth	9
Bour et al., 2018[45]	Norway	*Abra nitida*	Marine	Level 1	Adult	Metabolism	36
						Growth	9
Redondo et al., 2018[46]	Netherlands	*Asellus aquaticus*	Freshwater	Level 1	Adult	Behaviour	7
						Growth	7
						Survival	7
Xu et al., 2017[47]	Hong Kong	*Atactodea striata*	Marine	Level 1	Adult	Behaviour	6
						Metabolism	12
Lei et al., 2018[48]	China	*Caenorhabditis elegans*	Freshwater	Level 1	Adult	Growth	6
						Reproduction	12
						Survival	21
Watts et al., 2015[49]	UK	*Carcinus maenas*	Marine	Level 2	Adult	Behaviour	12
Watts et al., 2016[50]						Metabolism	15
						Growth	12
Cole et al., 2015[51]	UK	*Crassostrea gigas*	Marine	Level 1	Larvae	Behaviour	8
						Growth	2
Sussarellu et al., 2016[52]	France				Adult	Metabolism	1
						Growth	5
						Reproduction	3
Ziajahromi et al., 2018[53]	Australia	*Chironomus tepperi*	Freshwater	Level 1	Larvae	Growth	12
						Survival	4
Messinetti et al., 2018[54]	Italy	*Ciona robusta*	Marine	Level 1	Larvae	Growth	12

						Survival	4
Guilhermino et al., 2018[55]	Portugal	*Corbicula fluminea*	Freshwater	Level 1	Adult	Behaviour	8
Oliveira et al., 2018[56]	Portugal						
Rochman et al., 2017[2]	USA						
Bruck et al., 2018[57]	UK	*Echinogammarus marinus*	Marine	Level 2	Adult	Behaviour	18
						Growth	4
Bour et al., 2018[45]	Norway	*Ennucula tenuis*	Marine	Level 1	Adult	Metabolism	36
						Growth	9
Yu et al., 2018[58]	China	*Eriocheir sinensis*	Marine	Level 2	Adult	Metabolism	4
Blarer et al., 2016[59]	Switzerland	*Gammarus fossarum*	Freshwater	Level 1	Adult	Behaviour	24
Straub et al., 2017[60]						Growth	24
						Metabolism	24
Weber et al., 2018[61]	Germany	*Gammarus pulex*	Freshwater	Level 1	Juvenile	Behaviour	5
						Metabolism	10
						Growth	5
					Adult	Behaviour	12
Redondo et al., 2018[46]	Netherlands					Metabolism	10
						Growth	12
						Survival	7
Redondo et al., 2018[46]	USA	*Hyalella azteca*	Freshwater	Level 1	Juvenile	Growth	4
						Survival	8
Au et al., 2015[62]	Netherlands				Adult	Growth	10
						Survival	6
Hämer et al., 2014[63]	Germany	*Idotea emarginata*	Marine	Level 2	Juvenile	Behaviour	42
						Growth	6
Redondo et al., 2018[46]	Netherlands	*Lumbriculus variegatus*	Freshwater	Level 1	Adult	Behaviour	10
						Growth	7
						Reproduction	7

Browne et al., 2008[64]	UK	*Mytilus edulis*	Marine	Level 1	Larvae	Growth	39
Green et al., 2017[65]	Northern Ireland						
Green et al., 2019[66]	Northern Ireland						
Rist et al., 2019[67]	Denmark				Adult	Behaviour	14
van Cauwenberghe et al., 2015[44]	Belgium						
Woods et al., 2018[68]	USA					Metabolism	5
Capolupo et al., 2018[69]	Italy	*Mytilus galloprovincialis*	Marine	Level 1	Larvae	Behaviour	2
Beiras et al., 2018[70]	Spain					Growth	15
Détrée et al., 2018[71]	Chile				Adult	Growth	3
Devriese et al., 2017[72]	Belgium	*Nephrops norvegicus*	Marine	Level 2	Adult	Behaviour	5
Welden et al., 2016[73]	UK					Metabolism	2
						Growth	5
Green et al., 2017[65]	Northern Ireland	*Ostrea edulis*	Marine	Level 1	Adult	Behaviour	8
Green et al., 2016[74]						Metabolism	2
						Growth	4
Beiras et al., 2018[75]	Spain	*Paracentrotus lividus*	Marine	Level 1	Larvae	Growth	24
Beiras et al., 2019[70]							
Messinetti et al., 2018[54]	Italy						
Leung et al., 2018[76]	Hong Kong	*Perinereis aibuhitensis*	Marine	Level 1	Adult	Growth	14
Santana et al., 2018[77]	Brazil	*Perna perna*	Marine	Level 1	Adult	Behaviour	1
Rist et al., 2016[78]	Indonesia	*Perna viridis*	Marine	Level 1	Adult	Behaviour	2
						Metabolism	1
Gardon et al., 2018[79]	French Polynesia	*Pinctada margaritifera*	Marine	Level 1	Adult	Behaviour	6
						Metabolism	3
						Growth	6
Tosetto et al., 2016[80]	Australia	*Platorchestia smithi*	Marine	Level 1	Adult	Behaviour	2
						Growth	2
						Survival	2
Imhof et al., 2016[81]	Germany	*Potamopyrgus antipodarum*	Freshwater	Level 1	Adult	Growth	53

						Reproduction	6
						Survival	14
Redondo et al., 2018[46]	Netherlands	*Sphaerium corneum*	Freshwater	Level 1	Adult	Growth	7
						Survival	3
Kaposi et al., 2014[82]	Australia	*Tripneustes gratilla*	Marine	Level 1	Larvae	Behaviour	2
						Growth	8
						Survival	4
Redondo et al., 2018[46]	Netherlands	*Tubifex spp.*	Freshwater	Level 1	Adult	Behaviour	10
						Growth	7
						Survival	7

Table S3 **Summary of grouping and case studies number for concentration analysis**. Number of case studies reported for: per each medium (i.e. water, sediment and food); grouping factor depending on the technique adopted to measure microplastic; unit used to measure microplastic's concentration depending on the medium used for the experiment; final number of case studies after standardization to a common unit of measure per each of the three experiment's medium.

Medium		Group	Case studies	Unit used	Case studies	Standardized unit	Case studies
Water	937	Number	261	n MPs/ml	102	n MPs/ml	261
				n MPs/ 300ml	12		
				n MPs/individvidual	48		
				n MPs/L	99		
		Weight	676	µg MPs/ml	69	mg MPs/L	676
				µg MPs/L	51		
				mg MPs/L	556		
Sediment	234	Number	21	n MPs/ kg sediment	16	n MPs/kg sed	21
				n MPs/g sediment	5		
		Weight	90	mg MPs/Kg sediment	90	g MPs/Kg sed	213
		Percentage	123	%MPs/sediment.weight	123		
Food	205	Number	88	n MPs/mg food	23	n MPs/g food	88
				n MPs/g food	65		
		Number/Surface	1	n MPs/surface	1	removed	1
		Percentage	45	%MPs/mg food	45	g MPs/kg food	45
		Weight	71	mg MPs/g food	45		71
				mg MPs/g food	8		

				g MPs/kg food	18		
NA	97						

Table S4 **Summary of model with continuous factor**. Result for the meta-regression models analyzing effect size and microplastic concentration (expressed in different unit). Both results for complete and without outliers (out.rm) model are reported. Analysis conducted with mixed-effects model, using the rma.mv function of the metaphor package in R, including study Id and functional trait as random factor. Significant results ($p \leq 0.05$) are indicated in bold.

Medium	Unit	Model	*p*-value	Correlation
Water	n MPs/liter	mod1– complete	**0.0103**	negative
		mod2 – out.rm1	**0.0176**	positive
		mod3 – out.rm2	0.1461	NS
	mg MPs/liter	mod1 – complete	0.1712	NS
		mod2 – out.mr	**0.0330**	positive
Sediment	n MPs/kg sediment	mod1 - complete	**< .0001**	positive
	g MPs/kg sediment	mod1 - complete	0.6835	NS
Food	n MPs/kg food	mod1 - complete	0.3263	NS
		mod2 – out.rm	0.2721	NS
	g MPs/g food	mod1 - complete	0.5467	NS
		mod2 – out.rm	0.4828	NS

REFERENCES

1. Critchell, K. & Hoogenboom, M. O. Effects of microplastic exposure on the body condition and behaviour of planktivorous reef fish (*Acanthochromis polyacanthus*). *PLoS One* **13**, e0193308 (2018).

2. Rochman C.M., Parnis J.M., Browne M.A., Serrato S., Reiner E.J., Robson M., Young T., Diamond M.L., T. S. J. Direct and indirect effects of different types of microplastics on freshwater prey (*Corbicula fluminea*) and their predator (*Acipenser transmontanus*). *PLoS One* (2017).

3. Naidoo, T. & Glassom, D. Decreased growth and survival in small juvenile fish, after chronic exposure to environmentally relevant concentrations of microplastic. *Mar. Pollut. Bull.* **145**, 254–259 (2019).

4. Jabeen, K., Li, B., Chen, Q., Su, L., Wu, C., Hollert, H., & Shi, H. Effects of virgin microplastics on goldfish (*Carassius auratus*). *Chemosphere* **213**, 323–332 (2018).

5. Choi, J. S., Jung, Y.-J., Hong, N.-H., Hong, S. H. & Park, J.-W. Toxicological effects of irregularly shaped and spherical microplastics in a marine teleost, the sheepshead minnow (*Cyprinodon variegatus*). *Mar. Pollut. Bull.* **129**, 231–240 (2018).

6. Xia, X., Sun, M., Zhou, M., Chang, Z. & Li, L. Polyvinyl chloride microplastics induce growth inhibition and oxidative stress in Cyprinus carpio var. larvae. *Sci. Total Environ.* **716**, 136479 (2020).

7. Chen, Q., Gundlach, M., Yang, S., Jiang, J., Velki, M., Yin, D., & Hollert, H. Quantitative investigation of the mechanisms of microplastics and nanoplastics toward zebrafish larvae locomotor activity. *Sci. Total Environ.* **584**, 1022–1031 (2017).

8. Malafaia, G., de Souza, A. M., Pereira, A. C., Goncalves, S., da Costa Araujo, A. P., Ribeiro, R. X., & Rocha, T. L. Barrier function of zebrafish embryonic chorions against microplastics and nanoplastics and its impact on embryo development. *J. Hazard. Mater.* **395**, 122621 (2020).

9. Karami, A., Groman, D. B., Wilson, S. P., Ismail, P. & Neela, V. K. Biomarker responses in zebrafish (*Danio rerio*) larvae exposed to pristine low-density polyethylene fragments. *Environ. Pollut.* **223**, 466–475 (2017).

10. Qiang, L. & Cheng, J. Exposure to microplastics decreases swimming competence in larval zebrafish (*Danio rerio*). *Ecotoxicol. Environ. Saf.* **176**, 226–233 (2019).

11. Malafaia, G., de Souza, A. M., Pereira, A. C., Goncalves, S., da Costa Araujo, A. P., Ribeiro, R. X., & Rocha, T. L. Developmental toxicity in zebrafish exposed to polyethylene microplastics under static and semi-static aquatic systems. *Sci. Total Environ.* **700**, 134867 (2020).

12. LeMoine, C. M., Kelleher, B. M., Lagarde, R., Northam, C., Elebute, O. O., & Cassone, B. J. Transcriptional effects of polyethylene microplastics ingestion in developing zebrafish (*Danio rerio*). *Environ. Pollut.* **243**, 591–600 (2018).

13. Qiao, R., Deng, Y., Zhang, S., Wolosker, M. B., Zhu, Q., Ren, H., & Zhang, Y. Accumulation of different shapes of microplastics initiates intestinal injury and gut microbiota dysbiosis in the gut of zebrafish. *Chemosphere* **236**, 124334 (2019).

14. Kim, S. W., Chae, Y., Kim, D. & An, Y.-J. Zebrafish can recognize microplastics as inedible materials: Quantitative evidence of ingestion behavior. *Sci. Total Environ.* **649**, 156–162 (2019).

15. Jin, Y., Xia, J., Pan, Z., Yang, J., Wang, W., & Fu, Z. Polystyrene microplastics induce microbiota

dysbiosis and inflammation in the gut of adult zebrafish. *Environ. Pollut.* **235**, 322–329 (2018).

16. Limonta, G. *et al.* Microplastics induce transcriptional changes, immune response and behavioral alterations in adult zebrafish. *Sci. Rep.* **9**, 1–11 (2019).

17. Zhao, Y., Bao, Z., Wan, Z., Fu, Z. & Jin, Y. Polystyrene microplastic exposure disturbs hepatic glycolipid metabolism at the physiological, biochemical, and transcriptomic levels in adult zebrafish. *Sci. Total Environ.* **710**, 136279 (2020).

18. Mazurais, D. *et al.* Evaluation of the impact of polyethylene microbeads ingestion in European sea bass (Dicentrarchus labrax) larvae. *Mar. Environ. Res.* **112**, 78–85 (2015).

19. Barboza, L. G. A., Vieira, L. R. & Guilhermino, L. Single and combined effects of microplastics and mercury on juveniles of the European seabass (*Dicentrarchus labrax*): changes in behavioural responses and reduction of swimming velocity and resistance time. *Environ. Pollut.* **236**, 1014–1019 (2018).

20. Peda, C. *et al.* Intestinal alterations in European sea bass *Dicentrarchus labrax* (Linnaeus, 1758) exposed to microplastics: preliminary results. *Environ. Pollut.* **212**, 251–256 (2016).

21. Jinhui, S. *et al.* Effects of microplastics and attached heavy metals on growth, immunity, and heavy metal accumulation in the yellow seahorse, Hippocampus kuda Bleeker. *Mar. Pollut. Bull.* **149**, 110510 (2019).

22. Guven, O. *et al.* Microplastic does not magnify the acute effect of PAH pyrene on predatory performance of a tropical fish (*Lates calcarifer*). *Aquat. Toxicol.* **198**, 287–293 (2018).

23. Zhu, M., Chernick, M., Rittschof, D. & Hinton, D. E. Chronic dietary exposure to polystyrene microplastics in maturing Japanese medaka (*Oryzias latipes*). *Aquat. Toxicol.* **220**, 105396 (2020).

24. Chisada, S., Yoshida, M. & Karita, K. Ingestion of polyethylene microbeads affects the growth and reproduction of medaka, *Oryzias latipes*. *Environ. Pollut.* **254**, 113094 (2019).

25. Hu, L., Chernick, M., Lewis, A. M., Ferguson, P. L. & Hinton, D. E. Chronic microfiber exposure in adult Japanese medaka (*Oryzias latipes*). *PLoS One* **15**, e0229962 (2020).

26. Pannetier, P. *et al.* Toxicity assessment of pollutants sorbed on environmental microplastics collected on beaches: Part II-adverse effects on Japanese medaka early life stages. *Environ. Pollut.* **248**, 1098–1107 (2019).

27. Cong, Y. *et al.* Ingestion, egestion and post-exposure effects of polystyrene microspheres on marine medaka (*Oryzias melastigma*). *Chemosphere* **228**, 93–100 (2019).

28. Le Bihanic, F. *et al.* Organic contaminants sorbed to microplastics affect marine medaka fish early life stages development. *Mar. Pollut. Bull.* **154**, 111059 (2020).

29. Li, Y. *et al.* Low level of polystyrene microplastics decreases early developmental toxicity of phenanthrene on marine medaka (*Oryzias melastigma*). *J. Hazard. Mater.* **385**, 121586 (2020).

30. Wang, J. *et al.* Polystyrene microplastics cause tissue damages, sex-specific reproductive disruption and transgenerational effects in marine medaka (*Oryzias melastigma*). *Environ. Pollut.* **254**, 113024 (2019).

31. Lönnstedt, O. M. & Eklöv, P. Environmentally relevant concentrations of microplastic particles influence larval fish ecology. *Science (80-.).* **352**, 1213–1216 (2016).

32. Malinich, T. D., Chou, N., Sepúlveda, M. S. & Höök, T. O. No evidence of microplastic impacts on

consumption or growth of larval *Pimephales promelas*. *Environ. Toxicol. Chem.* **37**, 2912–2918 (2018).

33. de Sá, L. C., Luís, L. G. & Guilhermino, L. Effects of microplastics on juveniles of the common goby (*Pomatoschistus microps*): confusion with prey, reduction of the predatory performance and efficiency, and possible influence of developmental conditions. *Environ. Pollut.* **196**, 359–362 (2015).

34. Ferreira, P., Fonte, E., Soares, M. E., Carvalho, F. & Guilhermino, L. Effects of multi-stressors on juveniles of the marine fish *Pomatoschistus microps*: gold nanoparticles, microplastics and temperature. *Aquat. Toxicol.* **170**, 89–103 (2016).

35. Fonte, E., Ferreira, P. & Guilhermino, L. Temperature rise and microplastics interact with the toxicity of the antibiotic cefalexin to juveniles of the common goby (*Pomatoschistus microps*): post-exposure predatory behaviour, acetylcholinesterase activity and lipid peroxidation. *Aquat. Toxicol.* **180**, 173–185 (2016).

36. Luís, L. G., Ferreira, P., Fonte, E., Oliveira, M. & Guilhermino, L. Does the presence of microplastics influence the acute toxicity of chromium (VI) to early juveniles of the common goby (*Pomatoschistus microps*)? A study with juveniles from two wild estuarine populations. *Aquat. Toxicol.* **164**, 163–174 (2015).

37. Miranda, T., Vieira, L. R. & Guilhermino, L. Neurotoxicity, behavior, and lethal effects of cadmium, microplastics, and their mixtures on *Pomatoschistus microps* juveniles from two wild populations exposed under laboratory conditions—implications to environmental and human risk assessment. *Int. J. Environ. Res. Public Health* **16**, 2857 (2019).

38. Schmieg, H. *et al.* Polystyrene microplastics do not affect juvenile brown trout (*Salmo trutta* f. fario) or modulate effects of the pesticide methiocarb. *Environ. Sci. Eur.* **32**, 1–15 (2020).

39. Yin, L. *et al.* Impacts of polystyrene microplastics on the behavior and metabolism in a marine demersal teleost, black rockfish (*Sebastes schlegelii*). *J. Hazard. Mater.* **380**, 120861 (2019).

40. Yin, L., Chen, B., Xia, B., Shi, X. & Qu, K. Polystyrene microplastics alter the behavior, energy reserve and nutritional composition of marine jacopever (*Sebastes schlegelii*). *J. Hazard. Mater.* **360**, 97–105 (2018).

41. Espinosa, C., Cuesta, A. & Esteban, M. Á. Effects of dietary polyvinylchloride microparticles on general health, immune status and expression of several genes related to stress in gilthead seabream (*Sparus aurata* L.). *Fish Shellfish Immunol.* **68**, 251–259 (2017).

42. Wen, B. *et al.* Single and combined effects of microplastics and cadmium on the cadmium accumulation, antioxidant defence and innate immunity of the discus fish (*Symphysodon aequifasciatus*). *Environ. Pollut.* **243**, 462–471 (2018).

43. Green, D. S., Boots, B., Sigwart, J., Jiang, S. & Rocha, C. Effects of conventional and biodegradable microplastics on a marine ecosystem engineer (*Arenicola marina*) and sediment nutrient cycling. *Environ. Pollut.* (2016).

44. Van Cauwenberghe, L., Claessens, M., Vandegehuchte, M. B. & Janssen, C. R. Microplastics are taken up by mussels (*Mytilus edulis*) and lugworms (*Arenicola marina*) living in natural habitats. *Environ. Pollut.* (2015).

45. Bour, A., Haarr, A., Keiter, S. & Hylland, K. Environmentally relevant microplastic exposure affects sediment-dwelling bivalves. *Environ. Pollut.* (2018).

46. Redondo-Hasselerharm, P. E., Falahudin, D., Peeters, E. T. H. M. & Koelmans, A. A. Microplastic Effect Thresholds for Freshwater Benthic Macroinvertebrates. *Environ. Sci. Technol.* (2018).

47. Xu, X.-Y. *et al.* Microplastic ingestion reduces energy intake in the clam *Atactodea striata*. *Mar. Pollut. Bull.* (2017).

48. Lei, L. *et al.* Microplastic particles cause intestinal damage and other adverse effects in zebrafish Danio rerio and nematode *Caenorhabditis elegans*. *Sci. Total Environ.* (2018).

49. Watts, A. J. R., Urbina, M. A., Corr, S., Lewis, C. & Galloway, T. S. Ingestion of Plastic Microfibers by the Crab *Carcinus maenas* and Its Effect on Food Consumption and Energy Balance. *Environ. Sci. Technol.* (2015).

50. Watts, A. J. R. *et al.* Effect of Microplastic on the Gills of the Shore Crab *Carcinus maenas*. *Environ. Sci. Technol.* (2016).

51. Cole, M. & Galloway, T. S. Ingestion of Nanoplastics and Microplastics by Pacific Oyster Larvae. *Environ. Sci. Technol.* (2015).

52. Sussarellu, R. *et al.* Oyster reproduction is affected by exposure to polystyrene microplastics. *Proc. Natl. Acad. Sci. U. S. A.* (2016).

53. Ziajahromi, S., Kumar, A., Neale, P. A. & Leusch, F. D. L. Environmentally relevant concentrations of polyethylene microplastics negatively impact the survival, growth and emergence of sediment-dwelling invertebrates. *Environ. Pollut.* (2018).

54. Messinetti, S., Mercurio, S., Parolini, M., Sugni, M. & Pennati, R. Effects of polystyrene microplastics on early stages of two marine invertebrates with different feeding strategies. *Environ. Pollut.* (2018).

55. Guilhermino, L. *et al.* Uptake and effects of the antimicrobial florfenicol, microplastics and their mixtures on freshwater exotic invasive bivalve *Corbicula fluminea*. *Sci. Total Environ.* (2018).

56. Oliveira, P. *et al.* Effects of microplastics and mercury in the freshwater bivalve *Corbicula fluminea* (Müller, 1774): Filtration rate, biochemical biomarkers and mercury bioconcentration. *Ecotoxicol. Environ. Saf.* **164**, 155–163 (2018).

57. Bruck, S. & Ford, A. T. Chronic ingestion of polystyrene microparticles in low doses has no effect on food consumption and growth to the intertidal amphipod *Echinogammarus marinus*? *Environ. Pollut.* (2018).

58. Yu, P. *et al.* Accumulation of polystyrene microplastics in juvenile Eriocheir sinensis and oxidative stress effects in the liver. *Aquat. Toxicol.* (2018).

59. Blarer, P. & Burkhardt-Holm, P. Microplastics affect assimilation efficiency in the freshwater amphipod *Gammarus fossarum*. *Environ. Sci. Pollut. Res.* (2016).

60. Straub, S., Hirsch, P. E. & Burkhardt-Holm, P. Biodegradable and petroleum-based microplastics do not differ in their ingestion and excretion but in their biological effects in a freshwater invertebrate *Gammarus fossarum*. *Int. J. Environ. Res. Public Health* (2017).

61. Weber, A., Scherer, C., Brennholt, N., Reifferscheid, G. & Wagner, M. PET microplastics do not negatively affect the survival, development, metabolism and feeding activity of the freshwater invertebrate *Gammarus pulex*. *Environ. Pollut.* (2018).

62. Au, S. Y., Bruce, T. F., Bridges, W. C. & Klaine, S. J. Responses of *Hyalella azteca* to acute and chronic microplastic exposures. *Environ. Toxicol. Chem.* (2015).

63. Hämer, J., Gutow, L., Köhler, A. & Saborowski, R. Fate of microplastics in the marine isopod I*dotea emarginata*. *Environ. Sci. Technol.* (2014).

64. Browne, M. A., Dissanayake, A., Galloway, T. S., Lowe, D. M. & Thompson, R. C. Ingested microscopic plastic translocates to the circulatory system of the mussel, *Mytilus edulis* (L.). *Environ. Sci. Technol.* (2008).

65. Green, D. S., Boots, B., O'Connor, N. E. & Thompson, R. Microplastics Affect the Ecological Functioning of an Important Biogenic Habitat. *Environ. Sci. Technol.* (2017).

66. Green, D. S., Colgan, T. J., Thompson, R. C. & Carolan, J. C. Exposure to microplastics reduces attachment strength and alters the haemolymph proteome of blue mussels (*Mytilus edulis*). *Environ. Pollut.* (2019).

67. Rist, S., Baun, A., Almeda, R. & Hartmann, N. B. Ingestion and effects of micro- and nanoplastics in blue mussel (*Mytilus edulis*) larvae. *Mar. Pollut. Bull.* (2019).

68. Woods, M. N., Stack, M. E., Fields, D. M., Shaw, S. D. & Matrai, P. A. Microplastic fiber uptake, ingestion, and egestion rates in the blue mussel (*Mytilus edulis*). *Mar. Pollut. Bull.* (2018).

69. Capolupo, M., Franzellitti, S., Valbonesi, P., Lanzas, C. S. & Fabbri, E. Uptake and transcriptional effects of polystyrene microplastics in larval stages of the Mediterranean mussel *Mytilus galloprovincialis*. *Environ. Pollut.* (2018).

70. Beiras, R., Tato, T. & López-Ibáñez, S. A 2-Tier standard method to test the toxicity of microplastics in marine water using Paracentrotus lividus and *Acartia clausi* larvae. *Environ. Toxicol. Chem.* (2019).

71. Détrée, C. & Gallardo-Escárate, C. Single and repetitive microplastics exposures induce immune system modulation and homeostasis alteration in the edible mussel *Mytilus galloprovincialis*. *Fish Shellfish Immunol.* (2018).

72. Devriese, L. I., De Witte, B., Vethaak, A. D., Hostens, K. & Leslie, H. A. Bioaccumulation of PCBs from microplastics in Norway lobster (*Nephrops norvegicus*): An experimental study. *Chemosphere* (2017).

73. Welden, N. A. C. & Cowie, P. R. Long-term microplastic retention causes reduced body condition in the langoustine, Nephrops norvegicus. *Environ. Pollut.* **218**, 895–900 (2016).

74. Green, D. S. Effects of microplastics on European flat oysters, *Ostrea edulis* and their associated benthic communities. *Environ. Pollut.* **216**, 95–103 (2016).

75. Beiras, R. *et al.* Ingestion and contact with polyethylene microplastics does not cause acute toxicity on marine zooplankton. *J. Hazard. Mater.* (2018).

76. Leung, J. & Chan, K. Y. K. Microplastics reduced posterior segment regeneration rate of the polychaete Perinereis aibuhitensis. *Mar. Pollut. Bull.* (2018).

77. Santana, M. F. M., Moreira, F. T., Pereira, C. D. S., Abessa, D. M. S. & Turra, A. Continuous Exposure to Microplastics Does Not Cause Physiological Effects in the Cultivated Mussel *Perna perna*. *Arch. Environ. Contam. Toxicol.* (2018).

78. Rist, S. E. *et al.* Suspended micro-sized PVC particles impair the performance and decrease survival in the Asian green mussel *Perna viridis*. *Mar. Pollut. Bull.* (2016).

79. Gardon, T., Reisser, C., Soyez, C., Quillien, V. & Le Moullac, G. Microplastics Affect Energy Balance and Gametogenesis in the Pearl Oyster *Pinctada margaritifera*. *Environ. Sci. Technol.* (2018).

80. Tosetto, L., Brown, C. & Williamson, J. E. Microplastics on beaches: ingestion and behavioural consequences for beachhoppers. *Mar. Biol.* (2016).

81. Imhof, H. K. & Laforsch, C. Hazardous or not – Are adult and juvenile individuals of *Potamopyrgus*

antipodarum affected by non-buoyant microplastic particles? *Environ. Pollut.* (2016).

82. Kaposi, K. L., Mos, B., Kelaher, B. P. & Dworjanyn, S. A. Ingestion of microplastic has limited impact on a marine larva. *Environ. Sci. Technol.* (2014).
